# Supplementary material for: Designing and Implementing a Novel Virtual Rounds Curriculum for Medical Students' Internal Medicine Clerkship During the COVID-19 Pandemic
Source: MedEdPORTAL. 2021 Mar 2;17:11106. doi: 10.15766/mep_2374-8265.11106 (PMC7970635; doi:10.15766/mep_2374-8265.11106)
Supplement: Supplementary file 1 — VR Curriculum Guide.docxVirtual Rounds Orientation Guide.docxDiagnostic Reasoning Terms and Pitfalls.docxStudent Survey.docxTele-instructor Survey.docx [file mep_2374-8265.11106-s001.zip › A. VR Curriculum Guide.docx]

Virtual rounds

a medicine clerkship curriculum

# student objectives

- Virtually follow selected patients’ hospital courses in real time to understand how patients clinically evolve
- Become familiar with the format of daily rounds and understand how learners communicate with team members to care for patients
- Develop skills in identifying relevant electronic health record (EHR) data on hospitalized patients to create an assessment and plan
- Practice skills in delivering oral presentations, with a focus on assessment/plan
- Incorporate clinical reasoning into daily rounding discussions and presentations with tele-team (e.g., review elements such as schemas, problem representation)
- Receive and incorporate feedback from peers and attendings on above skills, and become more confident with these clinical skills in order to implement them during the clinical component of the clerkship later in the year

# Description

Summary: In Virtual Rounds, each clerkship student will virtually follow one patient on an inpatient wards team each week by reviewing the patient’s EHR and by listening in on the team’s daily patient presentation by audio. Students will then separately “round” with a tele-attending and clerkship tele-team later in the day.

All medicine clerkship students at this site will be assigned to one of eight medicine teaching wards teams (A-H) and follow a patient that the ward team’s acting intern (fourth-year medical student) is following. Priority will be given to assigning patients that are newly admitted (on call day or as holdover admission). Virtual Rounds will occur three times a week on consecutive days, for two weeks.

Students will assume a virtual student role that is focused solely on their learning; this role does NOT include direct patient care.

Tele-team structure: 3-4 clerkship students will be assigned to one “tele-attending,” an internal medicine attending focused on the education of these students. Efforts will be made to keep this attending the same for the entire 3-day span. If available, this team may also consist of tele-residents and tele-MS4s who will serve as teaching assistants on this virtual team.

Daily structure: A basic outline of the Virtual Rounds schedule can be found below (Figure 1). Upon assignment of a patient on the day before or morning of virtual rounds, a student is responsible for reviewing the EHR prior to the wards team rounds in the AM, and preparing an assessment and plan OR a SOAP presentation for the day based on the available and relevant EHR data (encouraged to spend about 30 minutes). Right before it is time for the wards team’s acting intern to present this patient, the acting intern should connect with their assigned virtual MS3 via telephone speaker or commercial videoconferencing software (such as Zoom^TM^, Microsoft Teams^TM^, FaceTime^TM^, etc.). The MS3 student will listen to the acting intern (or intern) synthesize an assessment and plan, and additionally will listen to the team’s discussion and teaching points relevant to this patient. The MS3 student will not be involved in direct patient care and will not be presenting the patient to the wards team, nor will they be communicating directly with the patient due to limited resources and bandwidth at this time. They can end the call after this patient.

In the late morning or afternoon, MS3 students will meet via videonferencing with their tele-attending and tele-team to “round” and present their patients (1 hour). The educational focus and goals of tele-rounding may vary each day, ranging from development of a problem representation and differential diagnosis to practice of oral presentations to teaching about diagnosis and management of a specific diagnosis related to assigned patient.

**Figure 1. Basic outline of Virtual Rounds schedule**


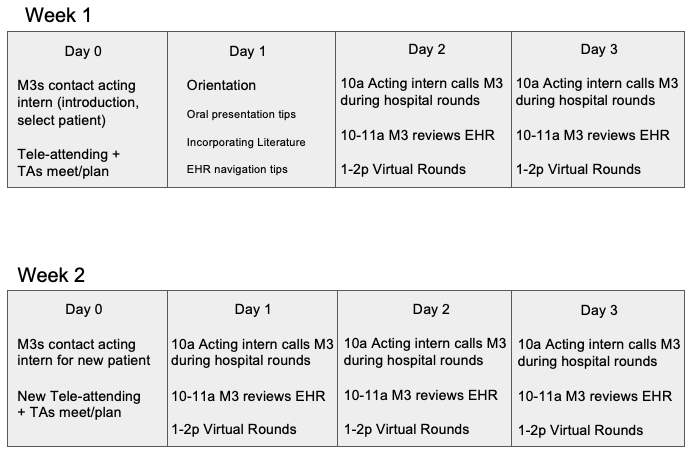


**Specific roles: *Please skip to the section that pertains to your role in Virtual Rounds.***

**Virtual MS3 (Virtual clerkship student)**:

- Before hospital rounds:
  - Check in (email) with the acting intern on your assigned wards team at the beginning of the week to select, together, one of your acting intern’s patients to “follow”—**please do so no later than Monday!** Make sure to exchange phone numbers so that the acting intern can reach you during morning rounds. A **sample introductory email template** for you to send to your acting intern is included below in the Appendix.
  - In a secure email, let your tele-team know which patient you will be following (provide the patient’s medical record number).
  - Once you have a new patient, read through their H&P and subsequent hospital course to understand where they are currently in their hospitalization (~15-30 min, can do the night before virtual rounds).
  - Spend no more than 60min each morning of virtual rounds (3 days/week) “pre-rounding” and preparing your presentation. First day will be orientation, but practicing will be helpful. Use this time to review patient’s 24hr events, labs, vitals, other diagnostic workup results. As best as possible without communicating with or examining the patient, prepare the A/P section of the oral presentation for the day. You may find it helpful to use resources such as UpToDate, PubMed Literature Searches, Infectious Disease Guidelines, etc. It helps to write down your Assessment/Plan (A/P), and then practice it verbally.
  - If your assigned patient has had imaging (such as a CXR) or other studies such as an EKG, practice reading and interpreting these on your own
- During hospital rounds:
  - Be prepared for the acting intern (or intern) to call/videoconference you during their presentation of your shared patient to the wards team. Listen in to how your acting intern crafts the oral presentation on your shared patient and learn from the ensuing team discussion.
  - In addition to medical management of the patient, make note of other important aspects of a patient’s hospital course (such as interprofessional communication that has taken place- PT/OT, or tasks performed by the acting intern that help with discharge planning).
- Before Virtual Rounds:
  - Tele-team (led by the tele-attending/assistant) will be in touch regarding daily timing of virtual rounds (60 min)- this can be anywhere from later in the morning to afternoon (the time will not conflict with your afternoon small group didactics). Be prepared to discuss problem representation and differential diagnosis, and to give an oral presentation for your patient.
  - At the end of your chart review (either the night before your virtual rounds or after your morning pre-rounds), try to research a relevant topic using UpToDate and the medical literature (on disease pathology, management, etc.). You can add the information you learn to your presentation and discuss evidence-based medicine with your attending, or use it to identify gaps in your knowledge that you would like to learn more about. Only do this if pre-rounding has taken less than 1 hour.
- During Virtual Rounds:
  - Present a <5-minute SOAP-style or just Assessment and Plan presentation to your tele-attending and peers (your team will set the goal for the day ahead of time). We understand you might not have much information in the subjective and physical exam portions of your presentation, so focus on interpreting existing data and constructing a well-thought out assessment and plan section. Do NOT simply copy the acting intern’s note; create your own note without reading the acting intern’s note for the day.
  - Bring up topics you researched and learned about, in addition to topics you are unfamiliar with and would like to learn more about (such as disease pathology, management, relevant imaging/EKG findings).
  - The goal is to practice your presentation skills and come up with interesting topics for discussion and learning that will help prepare you for your clinical rotations later this year.

**Acting intern:**

- Before rounds:
  - Your team’s MS3 will email you (Monday at the latest) to work together to select one patient that you are already following that would be an appropriate patient for them to follow along for their Virtual Rounds. Please note that the virtual clerkship students will NOT be participating in direct patient care.
  - Priority should be given to assigning patients that are recently admitted and have active medical problems. Ideally, the MS3 will follow the same patient all week. If this patient gets discharged, please guide the student in selecting a new patient to follow.
  - The “tele-attending” will include you in an email to the tele-team by way of introduction, and to offer themselves as a resource in case you have questions, such as needing advice on which patient would be best for the MS3 to virtually follow.
  - If you have your day off during one of the virtual rounding days, please email the clerkship leaders to discuss whether it is feasible for the MS3 to listen in to rounds with another team member coordinating that day.
- During rounds:
  - On morning rounds 3x/week (your student or the tele-attending will tell you which days they have Virtual Rounds), a few minutes before you will be presenting the shared patient on rounds, please either call or videoconference with the MS3 (can use phone or video tablet if available) so that they can listen in to your presentation and any discussion/teaching points on this case. Please end call after this.
- After rounds:
  - While we definitely appreciate that some acting interns will want to debrief patient cases with their MS3's and help teach them more about the case (e.g., emailing them clinical pearls, sending an end-of-day update of significant clinical events), this is NOT required. If you do have time for this, then that is great! We understand you have a demanding workload as acting interns that requires a lot of time and attention, and our request to help facilitate the education of MS3's should not compromise your clinical work. If you feel like you are not able to coordinate patient case assignments and videoconferencing times with your MS3, please let us know.

**In-person hospital team’s resident and attending:**

- Please be aware of this Virtual Rounds curriculum that allows MS3s to gain the skills as mentioned above. Each virtual student is assigned a team, Team A-H, similar to a “usual” clerkship. There are no specific tasks for the wards residents or attendings aside from allowing the acting intern to facilitate the MS3 listening in to their rounding presentation and subsequent discussions on their one shared patient. The acting intern will contact your virtual MS3 by phone or videoconferencing a few minutes before the acting intern’s presentation and will end the call after the presentation/discussion.
- Again, the MS3 is NOT involved in any direct patient care for this patient.
- If there are any teaching didactics later in the day on a topic related to this patient, feel free to let the MS3 know about this additional opportunity for learning (or have your acting intern reach out). Alternatively, if you have any teaching that you are doing electronically (e.g., sending articles or teaching points by email), please include the MS3 on your email chain as the MS3 will be enthusiastic about learning from you in this way.

**Tele-team attending:**

- Before Virtual Rounds:
  - Choose the dates and times for your week of Virtual Rounds. Virtual Rounds will be 1 hour/day (late morning or in afternoon), 3 days/week for Week 1 and Week 2- these should not conflict with students’ early afternoon didactics or resident noon conference. Choosing 3 consecutive days is preferable but not mandatory.
  - Email your virtual MS3s and tele-assistants (TAs) at the beginning of the week to introduce yourself. Inform them of the dates and times you will be leading Virtual Rounds and set the agenda for specific learning goals of rounds for each day (determine learning goals either on your own or with student feedback of what they would like to focus on). Students appreciate specific learning goals for the day for the entire team. There may be days you ask them to only prepare the A/P, and days you ask them to prepare the entire SOAP presentation. Note that even though MS3s often voice a strong desire to focus on disease management, we have emphasized the objectives of more "basic" skills that they often have not mastered, like forming a problem representation, prioritizing a problem list, appropriate differentials, oral presentation skills, constructing illness scripts, and interpreting diagnostic findings.
  - Please connect early with your TAs (tele-resident and tele-MS4s) to decide how you'd like to run rounds. It is completely up to your team to decide how to split up the teaching! From feedback, having the tele-resident "lead" rounds similar to real wards seemed most successful, and we encourage tele-residents and tele-MS4’s to coordinate teaching details together. Day 1 of Week 1 will be Orientation Day (see separate attachment).
  - Be available to acting intern if there are questions regarding which patient may be best for MS3 to follow.
  - If time allows, quickly review patients’ EMRs before Virtual Rounds that day, though this is not mandatory. Add your MS3s’ patients to your own APEX list. They should be emailing the medical record number to you once they have chosen a patient.
  - Optional: exchange phone numbers between MS3s and TAs to set up a phone group chat to facilitate easy communication for days of Virtual Rounds. Can also set up a shared Google Docs page.
- During Virtual Rounds:
  - Many tele-teams have expressed that they enjoy “Resident-led” Virtual Rounds, with residents leading the teaching and feedback, and attendings supplementing the discussion and providing additional learning resources. Feel free to come up with a rounding system that works best for your tele-team.
    - An example: MS4 gives the initial feedback on oral presentation focusing on presentation structure and best practices. Then the resident supplements with feedback on the medical management and diagnostic reasoning discussed in the A&P. The attending moderates, keeps track of time, and provides any final teaching points and learning resources. At the end of the session, MS4 or Resident gives a 5 to 10-minute chalk talk on a relevant clinical topic.
  - Students will be ready to present their patients for the day; there is flexibility in what you want each day to focus on, but along with your TAs, provide feedback on whichever skills or goals are focused on that day.
  - Students will not have thorough subjective or physical exam components of their presentation, so focus on their interpretation of available data and subsequent clinical reasoning and disease management.
- After Virtual Rounds:
  - If your MS3 has brought up information from their chart review that has clinical relevance to be addressed urgently and does not clearly seem to be brought to the wards team attention, please reach out to the wards attending to touch base about this. We do not expect this to occur often, but wanted to ensure students talked to you about anything that needs to be addressed.
  - We encourage use of email or a shared document to share educational materials to promote further learning (such as relevant papers or diagnostic schemas)

**Tele-team Resident teaching assistants:**

- Tele-attending will touch base with you at the beginning of the week to discuss structure of week and timing of virtual rounds for each day. Virtual rounds will be 1hr/day, 3 days/week for Week 1 and Week 2 of each EMLR block.
- Week 1 Day 1 will be an “Orientation Day” that you should lead parts of (see separate attachment), including screen sharing an EHR screen for tips on how to efficiently pre-round.
- Please connect early with your MS4 TA to talk about their teaching goals and decide how you'd like to run rounds. It is completely up to your team to decide how to split up the teaching! From student and faculty feedback, having the resident "lead" rounds similar to real wards seemed most successful!
  - An example: MS4 gives the initial feedback on oral presentation focusing on presentation structure and best practices. Then the resident supplements with feedback on the medical management and diagnostic reasoning discussed in the A&P. The attending moderates, keeps track of time, and provides any final teaching points and learning resources. At the end of the session, MS4 or Resident gives a 5 to 10-minute chalk talk on a relevant clinical topic.
  - Students really appreciate TA’s giving clinically relevant teaching such as “chalk-talks”. Discuss with your MS4 TA how you would like to split up this type of teaching. For example, MS4 might prepare 1-2 chalk talks a week, and on the other day the Resident can give an impromptu clinical talk.
- If time allows, review patient cases (3 MS3s’ patients) in EMR before Virtual Rounds. MS3 should be emailing you the patient’s MRN at the beginning of the week. Add them to your own APEX list.
- Assist in teaching points related to skills in developing differentials, problem representations, writing A/P notes, reviewing data (interpreting EKGs, labs, imaging) and oral presentations.

**Tele-team MS4 teaching assistants:**

- Tele-attending will touch base with you at the beginning of the week to discuss structure of week and timing of virtual rounds for each day. Virtual rounds will be 1hr/day, 3 days/week for Week 1 and Week 2 of each EMLR block.
- Week 1 Day 1 will be an “Orientation Day” that you should lead parts of (see separate attachment), including giving a best-practices SOAP presentation example.
- Please connect early with your resident and attending to decide how the team runs rounds. It is completely up to your team to decide how to split up the teaching! From student and faculty feedback, having the resident "lead" rounds similar to real wards seemed most successful!
  - An example: MS4 gives the initial feedback on oral presentation focusing on presentation structure and best practices. Then the resident supplements with feedback on the medical management and diagnostic reasoning discussed in the A&P. The attending moderates, keeps track of time, and provides any final teaching points and learning resources. At the end of the session, MS4 or Resident gives a 5 to 10-minute chalk talk on a relevant clinical topic.
- Discuss your goals for teaching with your Resident and attending. Here are some examples of different ways that prior MS4 TA’s have felt they were best able to contribute to Virtual Round teaching:
  - Tips on how to pre-round efficiently
  - Feedback on presentations that focus on structure, style, and best practices
  - Tips on how to include medical literature within presentations
  - Feedback on one-liners and basic diagnostic reasoning
  - Preparing 1-2 quick chalk talks on clinical topics every week
  - Systematic approach to interpreting labs, EKG or CXR
  - Emailing the group after Virtual Rounds with a summary of the learning points and learning resources or maintaining a Google Doc with important clinical pearls
- As you become more comfortable giving feedback on oral presentations and teaching clinical topics, you can talk to your Resident about taking on a larger teaching role, which may include providing more feedback on medical management and leading more impromptu teaching talks.

**Template email for MS3’s to send to acting interns:**

Hi __________

My name is ____ and I am a clerkship student assigned to your medicine team to follow a patient for our virtual rounds. As part of virtual rounds, I will chart review one of your patients this week to learn more about them and their hospital course. I will listen to your presentations and subsequent team discussions on this patient three days this week, but I will not be participating in any direct patient care for this patient.

It would be great if you could email me with the medical record number of one of your patients who you think would be interesting for me to follow this week. Ideally, this would be someone who was recently admitted and who has ongoing medical problems appropriate for my level of learning.

My phone number is _______. Do you mind sharing your number? Please let me know when your team starts rounds each day, and call/videoconference me into rounds right before you present so that I can listen to your presentation and the subsequent team discussion. We can end the call after this.

Thank you for your help in coordinating this learning experience for me.

Best,
