# Supplementary material for: Designing and Implementing a Novel Virtual Rounds Curriculum for Medical Students' Internal Medicine Clerkship During the COVID-19 Pandemic
Source: MedEdPORTAL. 2021 Mar 2;17:11106. doi: 10.15766/mep_2374-8265.11106 (PMC7970635; doi:10.15766/mep_2374-8265.11106)
Supplement: Supplementary file 1 — VR Curriculum Guide.docxVirtual Rounds Orientation Guide.docxDiagnostic Reasoning Terms and Pitfalls.docxStudent Survey.docxTele-instructor Survey.docx [file mep_2374-8265.11106-s001.zip › B. Virtual Rounds Orientation Guide.docx]

**Virtual Rounds Orientation Handout**

The following guide is meant to act as a framework for a tele-resident and tele-MS4 led orientation of three primary content areas on the first day of Virtual Rounds. The content was selected based upon direct feedback from students who participated in the first iteration of the Virtual Rounds curriculum. The three major content areas to review are:

- Developing a systematic approach to pre-rounding
- Delivering an effective SOAP-style oral presentation
- Incorporating relevant literature into the oral presentation

The guide below is provided as a framework for how to review these content areas at an MS3 level, but please feel free to deviate from the specific content and add in your own tips as you see fit. Our hope is that the full orientation day, like the other virtual rounding sessions, should last no more than 60 minutes. *Our suggestion for the order, timing, and role leadership of the orientation session is as follows, but again please feel free to deviate as you see fit*:

- Check-in and introductions led by attending (10 minutes)
- Setting goals/expectations for virtual rounds led by attending (10 minutes)
- Pre-rounding [screen sharing by resident] (10 minutes)
- SOAP-style presentation [key points and example SOAP presentation delivered by MS4] (10 minutes)
- Incorporating literature into presentation [key points and example delivered by MS4] (10 minutes)
- Wrap up led by attending (5 minutes)

Thank you again for your participation in Virtual Rounds!

**Please share your screen as you walk through your process of pre-rounding!**

**Note: Location of information within Epic-based systems italicized within parentheses following each row below**

**Pieces of information to collect when pre-rounding on a patient you have previously presented to the team (led by tele-resident):**

- Vital signs trends over past 24 hours (*Comprehensive tab*)
- In’s/Out’s (*Comprehensive tab*)
- Pertinent labs depending upon condition (*Results* *Review*)
  - Example: Cr/BUN in HF patient receiving diuretics
  - Example: WBC count in patient with infection
- Medication History (including PRN meds) over past 24 hours (*Index 🡪 Medication History*)
- Specific medication categories to check, as pertinent
  - Pain medications (*Pain tab)*
  - Insulin dosing (*Insulin tab*)
  - Antibiotics administered (*Fever/Antibiotics tab*)
- Notes from past 24 hours, especially from consultants (*Notes section*)
- Review history of pages (can often provide information about important events or potential overnight events)

**Pieces of information to collect when performing a “chart biopsy” for a patient who is admitted by a hospital team:**

- Past Hospitalizations History (*Chart review 🡪 Notes 🡪 “DC Summ” filter*)
- Admission H&P (*Notes*)
- +/- Information from outside hospitals, especially if patient receives majority of care at other healthcare institution (under a special tab)

Same sections as for patient who has been previously presented to team:

- Vital signs trends over past 24 hours (*Comprehensive tab*)
- I’s/O’s (*Comprehensive tab*)
- Pertinent labs depending upon condition (*Results* *Review*)
  - Example: Cr/BUN in HF patient you are diuresing
  - Example: WBC count in patient with infection
- Medication History (including PRN meds) over past 24 hours (*Index 🡪 Medication History*)
- Specific medications to check, as pertinent
  - Pain medications (*Pain tab)*
  - Insulin dosing (*Insulin tab*)
  - Antibiotics administered (*Fever/Antibiotics tab*)
- Notes from past 24 hours, especially from consultants (*Notes section*)
- Review history of pages (can often provide information about important events or potential overnight events)

**Key points about presentations (led by tele-MS4):**

- Conciseness and consistency to keep audience engaged
  - Note should be longer than presentation
  - Try to follow same order every time you present
  - Do not need to include all components of physical exam and labs in presentation
  - Do not include every problem a patient has; prioritize the problem list and only talk about problems you are actively managing that require team discussion
- Update problem representation to reflect clinical course
  - Provide most specific diagnostic name
  - Use phrases such as “worsening/stable/improving” when listing problems in A/P
- Include reason for continued hospitalization and requirements for hospital discharge

**Sample SOAP Oral Presentation for tele-MS4 to Model:**

“Ms. Jones is our 76-year-old woman on hospital day #2 currently being treated for suspected CAP, now on day 2/5 of IV ceftriaxone and PO doxycycline.

Overnight Events: None

**S:** Patient feels better than yesterday and is more aware of her situation. She states her breathing is improved, able to walk to the bathroom alone without SOB. Continued productive cough is unchanged. Denies fevers.

**O:** Vitals over last 24 hours:
Tmax 99.5F (Afebrile over past 24 hours)

BP 120-130s/60-80s

HR 70-80s

RR 16-23 (currently 16)

SpO2 90s% (now on room air from 2L nasal cannula)

Net In/Out for last 24hours: +135 mL

Physical Exam:

Gen: Well-appearing, A&O x 3, more interactive than yesterday,

Resp: no increased work of breathing on RA – improved; continued right base crackles, good air movement throughout

Remainder of exam unchanged from yesterday.

Pertinent Labs:

WBC 9.4 (down from 15.6 on admission)

Cr 1.3 (down from 1.8 on admission, but not yet at baseline of 0.7 – 0.8)

Blood cultures no growth to date x 2

RVP negative

Blood glucose = 100-125 on insulin sliding scale

No new imaging to report

**Assessment/Plan**:

Ms. Jones is a 76-year-old woman with controlled type 2 diabetes who presented with symptoms concerning for community-acquired pneumonia and who is now showing signs of clinical improvement on antibiotics.

**Problem #1: Community-Acquired Pneumonia** – Improving; consistent with bacterial pneumonia given the lobar opacification on her admission CXR and improvement of her leukocytosis and respiratory status on antibiotics. Plan to discharge tomorrow if patient remains on RA with stable vitals and if patient maintains adequate oxygenation upon ambulation

Diagnostic plan: No more workup is needed

Therapeutic plan:

- started IV ceftriaxone and PO doxycycline yesterday with plans to transition to PO doxycycline tomorrow in preparation for discharge

- Plan to complete a total of 5 days of antibiotics (currently day #2 of 5 of antibiotics)

- Ambulatory SpO2 prior to discharge

**Problem #2: Acute Kidney Injury** – Improving; Creatinine 1.8 on admission. Cr currently 1.3, baseline 0.7-0.8; likely secondary to pre-renal etiology given BUN:Creatinine > 20 and rapid improvement with fluids. Less likely obstruction or intrinsic renal disease.

Diagnostic plan: Given clinical improvement with fluids, no need to workup another cause of AKI

Therapeutic plan:

- hold hydrochlorothiazide

- encourage oral fluid intake

- strict I/O’s, goal net even

**Problem #3: Acute Encephalopathy/Altered Mental Status** – Resolved; likely secondary to acute infection.

- continue delirium precautions

**All other chronic problems are stable**.”

**~~Type 2 Diabetes~~** ~~– Chronic; Controlled only on metformin at home. Last A1c 6.1 in 10/2018. BGs currently well controlled~~

~~- hold metformin in setting of AKI~~

~~- continue insulin sliding scale~~

**~~Hypertension~~** ~~– Chronic; currently normotensive~~

~~- hold home amlodipine and hydrochlorothiazide in setting of sepsis~~

~~- consider resuming these medications once infection is under control and when AKI resolves~~

**Integrating literature into presentation (tele-MS4)**

1. Actual clinical question that prompted lit search
2. Title, publishing journal, year of publication and basic description of paper you found
3. Summary of key results pertinent to question of interest
4. Bring results back to your patient to inform what would be the most appropriate clinical decision

Example: “One question regarding the treatment of Ms. Jones is the duration of antibiotic treatment needed to adequately treat community-acquired pneumonia. To address this question, I performed a literature review and found a 2018 meta-analysis entitled *Systemic Review and Meta-analysis of the Efficacy of Short-Course Antibiotic Treatments for Community-Acquired Pneumonia in Adults* in the journal *Antimicrobial Agents and Chemotherapy*. The meta-analysis compared clinical outcomes for patients treated with </= 6 days of antibiotics as compared to those receiving >/= 7 days. The study captured 19 randomized clinical trials with a combined N of 4,069. The authors found that there was no significant difference in clinical cure between the groups even after controlling for treatment setting and severity of pneumonia. Importantly, they found that patients receiving </= 6 days of antibiotics had fewer serious adverse events and lower mortality compared to patients receiving >/= 7 days of antibiotics. Based on these findings, I am in favor of a 5 day antibiotic course for Ms. Jones assuming that she remains afebrile, continues to sat well on room air and remains clinically stable.”

**Pro tip:** In order to efficiently search the literature for high-quality papers, I would recommend two strategies:

1. Type “X clinical guidelines” where X is the disease process or clinical question, and then look at most up to date guidelines and literature cited within guidelines
2. Review references cited on UptoDate
