# Supplementary material for: Designing and Implementing a Novel Virtual Rounds Curriculum for Medical Students' Internal Medicine Clerkship During the COVID-19 Pandemic
Source: MedEdPORTAL. 2021 Mar 2;17:11106. doi: 10.15766/mep_2374-8265.11106 (PMC7970635; doi:10.15766/mep_2374-8265.11106)
Supplement: Supplementary file 1 — VR Curriculum Guide.docxVirtual Rounds Orientation Guide.docxDiagnostic Reasoning Terms and Pitfalls.docxStudent Survey.docxTele-instructor Survey.docx [file mep_2374-8265.11106-s001.zip › C. Diagnostic Reasoning Terms and Pitfalls.docx]

**Diagnostic Reasoning Concepts/Pitfalls**

**Diagnostic Reasoning Terms:**

1. **Schema:** A systematic approach to organizing the diagnostic approach to a given clinical symptom/problem that can help guide the history and physical, as well as the generation of a differential diagnosis.

- The first step is often the generation of broad categories or “buckets”. May be based on mechanistic thinking/pathophysiology or categories of disease. *Often unique to an individual clinician, based on their experience as well as learning/thinking preferences*
- Examples: [insert your own examples relevant to your specialty]
  - - 1. Hyperbilirubinemia = increased production, decreased conjugation, decreased excretion
      2. New rash = infectious, allergic, inflammatory/autoimmune
      3. Cyanosis = Cardiac mixing lesion vs severe pulmonary hypoxia vs methemoglobinemia
- Expert clinicians may not even realize they are doing this – **externalizing this process is critical to teaching** diagnostic reasoning to early learners
- Initial questions/diagnostics should attempt to **direct attention to one or eliminate another broad category**. Each category can then be further subdivided to allow for more nuanced diagnostics (eg: within “increased production bilirubin” would be: autoimmune hemolysis, traumatic hemolysis, high Hct, fragile RBC, etc)

1. **Problem Representation (PR):** Succinct one-sentence summary of a patient’s story ( “one-liner”; “summary statement”).

- **GENERAL FORMULA:** “This is a (*age, gender*) with history of (*relevant pmhx/risk fx*) presenting with (*relevant sx/signs*) found to have (*PE/diagnostics*) which is most consistent with (*prioritized ddx*)”
- **THIS TERM IS ONE OF THE MOST CONFUSING as it can be used in many different ways, and students are often confused about what/how much information to include. **
- Should include only the MOST RELEVANT aspects of the patient’s **risk factors, symptoms and time course**, and sometimes the most likely diagnosis. Allows experienced clinicians to use pattern recognition to **quickly develop a mental picture of the patient**, as well as generate a differential diagnosis and/or plan
- Uses **“processed terms”** – translation of patient’s story into medical terms, allowing comparison to mental maps
  - Examples: “coming and going” = intermittent; “involving the face, trunk, back, arms and legs” = generalized; “cough, runny nose and congestion” = “URI symptoms”
- Examples relevant to inpatient medicine:
  - **Setting the stage for a SOAP presentation** *- “NR is our 65 yo man with a history of CAD, CHF and COPD admitted yesterday with …”*
  - **The “one-liner”: a quick way of communicating the key aspects of a patient’s current status:** *“TJ is our 45 year old F with SLE c/b lupus nephritis s/p kidney transplant who p/w new fevers and an AKI*
  - **As the first sentence of the assessment** - *“Mr. Johnson is a previously healthy 75 M admitted last night with altered mental status, who was euvolemic on exam and found to have a Na+ of 125, now being managed with salt tablets and fluid restriction with improvement in Na+ levels and symptoms, most consistent with a diagnosis of symptomatic hyponatremia.*

1. **Illness Script:** A way of organizing the **most typical presentation/characteristics of a particular disease** into a table.

- Can be used to compare diseases that present similarly (eg CAD vs Aortic dissection vs pericarditis as a cause of chest pain) or compare a given patient to the typical patient with the proposed condition.
- Students are encouraged to create them for new diseases they encounter in their clinical rotations

**EXAMPLE ILLNESS SCRIPT TABLE**

***Symptom or presenting condition (eg: “Sudden loss of consciousness”)***

| DDx | Predisposing Factors (“Who”) | Clinical Features (“What”) | Time Course (“When”) | Pathophysiology (“Why”) |
| --- | --- | --- | --- | --- |
| Seizure | Variable depending upon the cause; medications, metabolic disturbances, alcohol withdrawal, hyperthyroidism, drugs/toxins | Transient loss of consciousness, urinary incontinence, prolonged period of recovery associated confusion, aura (abnormal visual or olfactory sensations before), NO orthostatics or prodrome (nausea, sweating, lightheadedness) | Hyperacute/ acute with short duration | Disordered firing of neurons |
| Vasovagal Response | Prolonged standing, acute stressors (including sight of blood), severe pain, Valsalva (coughing, defecation, urination) | Preceded by prodrome (nausea, sweating, lightheadedness), post-symptoms: fatigue, but no confusion | Hyperacute/ acute | Increased vagal tone leads to bradycardia and peripheral vasodilation |
| Syncope due to arrhythmia (Long QT) | Variable depending upon the cause; in the case of long QT, it can be congenital so there may be family history of arrhythmias or sudden death at a young age;  CAD or symptoms of ischemia [particularly when dealing with older adults] | Palpitations, but otherwise minimal preceding symptoms; common to have injury; exertion can trigger syncope; quick recovery with minimal confusion following | Hyperacute/acute | Abnormal electrical activity leads to decreased cardiac output and transient hypotension, leading to decreased blood flow to the brain and syncope |

**ILLNESS SCRIPT TEMPLATE**

| DDx | Predisposing Factors (“Who”) | Clinical Features (“What”) | Time Course (“When”) | Pathophysiology (“Why”) |
| --- | --- | --- | --- | --- |
|  |  |  |  |  |
|  |  |  |  |  |
|  |  |  |  |  |
