# Supplementary material for: Designing and Implementing a Novel Virtual Rounds Curriculum for Medical Students' Internal Medicine Clerkship During the COVID-19 Pandemic
Source: MedEdPORTAL. 2021 Mar 2;17:11106. doi: 10.15766/mep_2374-8265.11106 (PMC7970635; doi:10.15766/mep_2374-8265.11106)
Supplement: Supplementary file 1 — VR Curriculum Guide.docxVirtual Rounds Orientation Guide.docxDiagnostic Reasoning Terms and Pitfalls.docxStudent Survey.docxTele-instructor Survey.docx [file mep_2374-8265.11106-s001.zip › D. Student Survey.docx]

Virtual Rounds Student Survey

We appreciate your feedback on the Virtual Rounds curriculum. Please use this survey ONLY to give feedback on Virtual Rounds (pre-rounding, rounding, and small groups). Please rate the usefulness of the following components of the Virtual Rounds Curriculum in preparing you for the clinical portion of your Medicine Clerkship.

1. Developing an Approach to Pre-rounding

|  | Not at all Useful (1) | Slightly Useful (2) | Moderately Useful (3) | Very Useful (4) | Extremely Useful (5) |
| --- | --- | --- | --- | --- | --- |
| Practicing how to use the electronic health record to identify relevant patient data for rounds (1) |  |  |  |  |  |
| Developing an approach to chart reviewing (2) |  |  |  |  |  |
| Incorporating new labs/imaging/results into your presentation (3) |  |  |  |  |  |
| Incorporating resources, such as literature searches or Uptodate.com, into your presentation (4) |  |  |  |  |  |
| Overall usefulness of pre-rounding (5) |  |  |  |  |  |

2. Listening to Hospital Rounds

|  | Not at all Useful (1) | Slightly Useful (2) | Moderately Useful (3) | Very Useful (4) | Extremely Useful (5) |
| --- | --- | --- | --- | --- | --- |
| Listening to the acting intern or intern’s presentation |  |  |  |  |  |
| Listening to feedback on the acting intern or intern’s presentation |  |  |  |  |  |
| Exposure to patient-related discussions that occur on rounds |  |  |  |  |  |
| Overall usefulness of listening to hospital rounds |  |  |  |  |  |

3. Engaging in Virtual Rounds Small Group

|  | Not at all Useful (1) | Slightly Useful (2) | Moderately Useful (3) | Very Useful (4) | Extremely Useful (5) |
| --- | --- | --- | --- | --- | --- |
| Following a patient's clinical course |  |  |  |  |  |
| Giving SOAP or A+P presentations |  |  |  |  |  |
| Receiving feedback on oral presentations |  |  |  |  |  |
| Practicing how to incorporate feedback into subsequent oral presentations |  |  |  |  |  |
| Practicing skills in clinical reasoning (including problem representation, developing differentials) |  |  |  |  |  |
| Listening to oral presentations given by peers |  |  |  |  |  |
| Listening to feedback given to peers on their oral presentations |  |  |  |  |  |
| Advancing clinical knowledge |  |  |  |  |  |
| Overall usefulness of Virtual Rounds small group |  |  |  |  |  |

4. Compared to 3 weeks ago, after completing the Virtual Rounds Curriculum, how have the following changed:

|  | Much Worse (1) | Somewhat Worse (2) | About the Same (3) | Somewhat Better (4) | Much Better (5) |
| --- | --- | --- | --- | --- | --- |
| Your ability to preround |  |  |  |  |  |
| Your ability to give an oral presentation |  |  |  |  |  |
| Your ability to clinically reason |  |  |  |  |  |
| Your confidence (as you approach the clinical component of your Medicine Rotation) |  |  |  |  |  |

5. Please rate the quality of the following technology components

|  | Very Poor (1) | Poor (2) | Fair (3) | Good (4) | Excellent (5) |
| --- | --- | --- | --- | --- | --- |
| Ability to hear the acting intern or intern's wards presentation over the phone |  |  |  |  |  |
| Virtual environment (via Zoom) as a space that allows for achieving curriculum learning objectives |  |  |  |  |  |

6. What virtual learning resources were used by your small group? Mark all that apply:

- Screen Sharing Technology (i.e looking at literature, word documents, “chalk talks”, powerpoints together)
- Google Doc
- Evernote
- MD Calc
- UpToDate
- PubMed
- Clinical Problem Solvers Website
- Online Textbook
- Other (please list below) ________________________________________________

Q12 What aspects of the curriculum did you find most valuable and why?

________________________________________________________________

________________________________________________________________

________________________________________________________________

________________________________________________________________

________________________________________________________________

Q13 How did following a real patient virtually compare to learning from resources with prepared cases (e.g. Case Files, Medical Podcasts, NEJM Interactive Cases, etc.)?

________________________________________________________________

________________________________________________________________

________________________________________________________________

________________________________________________________________

________________________________________________________________

Q14 How could the curriculum be improved?

________________________________________________________________

________________________________________________________________

________________________________________________________________

________________________________________________________________

________________________________________________________________
