# Supplementary material for: Designing and Implementing a Novel Virtual Rounds Curriculum for Medical Students' Internal Medicine Clerkship During the COVID-19 Pandemic
Source: MedEdPORTAL. 2021 Mar 2;17:11106. doi: 10.15766/mep_2374-8265.11106 (PMC7970635; doi:10.15766/mep_2374-8265.11106)
Supplement: Supplementary file 1 — VR Curriculum Guide.docxVirtual Rounds Orientation Guide.docxDiagnostic Reasoning Terms and Pitfalls.docxStudent Survey.docxTele-instructor Survey.docx [file mep_2374-8265.11106-s001.zip › E. Tele-instructor Survey.docx]

Virtual Rounds Teacher Survey

Thank you for volunteering to help facilitate the continued learning of 3rd year medical students while they are temporarily learning outside of the hospital. We appreciate the time and effort you put into teaching and furthering medical education. Please provide feedback on your experience facilitating and teaching Virtual Rounds to help us improve the course.

What was your role in Virtual Rounds?

- Attending
- Resident TA
- MS4 TA

Compared to hospital rounds, how would you rate your experience in facilitating/teaching Virtual Rounds with regards to the following:

|  | Much Worse (1) | Somewhat Worse (2) | About the Same (3) | Somewhat Better (4) | Much Better (5) |
| --- | --- | --- | --- | --- | --- |
| Opportunities to provide feedback on oral presentation skills |  |  |  |  |  |
| Opportunities to provide feedback on medical management discussed in A&P |  |  |  |  |  |
| Opportunities to provide medical student level clinical teaching |  |  |  |  |  |
| Your overall ability to teach (comment below with details) |  |  |  |  |  |

Compared to the first oral presentations students gave on Virtual Rounds, how would you rate their abilities by the end of your time together on Virtual Rounds:

|  | Much Worse (1) | Somewhat Worse (2) | About the same (3) | Somewhat Better (4) | Much better (5) |
| --- | --- | --- | --- | --- | --- |
| Organization of oral presentation |  |  |  |  |  |
| Efficiency of oral presentation |  |  |  |  |  |
| Diagnostic reasoning conveyed in oral presentation |  |  |  |  |  |
| Clinical knowledge conveyed in oral presentation |  |  |  |  |  |
| Confidence in giving oral presentation |  |  |  |  |  |

Were there learning opportunities available through Virtual Rounds that would not have been available during hospital rounds (e.g increased time for feedback, pre-rounding on a shared computer screen or looking up medical literature in real time)?

________________________________________________________________

________________________________________________________________

________________________________________________________________

________________________________________________________________

________________________________________________________________

Do you think there is a role for Virtual Rounds to remain part of the Medicine Clerkship even when students return to hospital wards?

- Yes
- Maybe
- No

How likely would it be for you to volunteer to teach/facilitate Virtual Rounds in the future, if it fit into your schedule?

- Very likely
- Likely
- Neither likely nor unlikely
- Unlikely
- Very unlikely

Did participating in Virtual Rounds enhance your interests in medical education? If yes, how?

- Yes ________________________________________________
- No

Did you personally benefit from facilitating Virtual Rounds? If yes, how?

- Yes ________________________________________________
- No

What aspects of Virtual Rounds did you most enjoy?

________________________________________________________________

________________________________________________________________

________________________________________________________________

________________________________________________________________

________________________________________________________________

How could Virtual Rounds be improved?

________________________________________________________________

________________________________________________________________

________________________________________________________________

________________________________________________________________

________________________________________________________________

Do you have any additional comments on how Virtual Rounds compared to Hospital Rounds?

________________________________________________________________

________________________________________________________________

________________________________________________________________

________________________________________________________________

________________________________________________________________
