# Supplementary material for: Designing and Implementing a Novel Virtual Rounds Curriculum for Medical Students' Internal Medicine Clerkship During the COVID-19 Pandemic
Source: MedEdPORTAL. 2021 Mar 2;17:11106. doi: 10.15766/mep_2374-8265.11106 (PMC7970635; doi:10.15766/mep_2374-8265.11106)
Supplement: Supplementary file 1 — VR Curriculum Guide.docxVirtual Rounds Orientation Guide.docxDiagnostic Reasoning Terms and Pitfalls.docxStudent Survey.docxTele-instructor Survey.docx [file mep_2374-8265.11106-s001.zip › _Educational_Summary_Report_11106.pdf]

MedEdPORTAL® The AAMC Journal of Teaching and Learning Resources

Original Publication Open Access

Designing and Implementing a Novel Virtual Rounds Curriculum for Medical Students' Internal Medicine Clerkship During the COVID-19 Pandemic

Smrithi Sukumar*, Adam Zakaria, Cindy J. Lai, MD, Matthew Sakumoto, MD, Raman Khanna, MD, MAS, Nancy Choi, MD

*Corresponding author: smrithi.sukumar@ucsf.edu

Abstract

Introduction: During the COVID-19 pandemic, third-year medical students were temporarily unable to participate in onsite clinical activities. We identified the curricular components of an internal medicine (IM) clerkship that would be compromised if students learned solely from online didactics, case studies, and simulations (i.e., prerounding, oral presentations, diagnostic reasoning, and medical management discussions). Using these guiding principles, we created a virtual rounds (VR) curriculum to provide IM clerkship students with clinical exposure during a virtual learning period. **Methods:** Held three times a week for 2 weeks, VR consisted of three curricular components. First, clerkship students prerounded on an assigned hospitalized patient by remotely accessing the electronic health record and calling into hospital rounds. Second, each student prepared an oral presentation on their assigned patient. Third, using videoconferencing, students delivered these oral presentations to telemedicine VR small groups consisting of three to four students and three tele-instructors. Tele-instructors then provided feedback on oral presentations and taught clinical concepts. We assessed the effectiveness of VR by anonymously surveying students and tele-instructors. **Results:** Twenty-nine students and 34 volunteer tele-instructors participated in VR over four blocks. A majority of students felt VR improved their prerounding abilities (86%), oral presentation abilities (93%), and clinical reasoning skills (62%). All students found small group to be useful. **Discussion:** VR allowed students to practice rounding skills in a supportive team-based setting. The lessons learned from its implementation could facilitate education during future pandemics and could also supplement in-person clerkship education.

Keywords
COVID-19, Distance Learning, Clinical Medical Education, Clerkship, Internal Medicine, Virtual Rounds, Telemedicine, Chart Review, Clinical Teaching/Bedside Teaching, Hospital Medicine, Virtual Learning

Educational Objectives

By the end of this activity, learners will be able to:

1. Virtually follow the course of at least one hospitalized patient on the internal medicine service per week to understand their clinical evolution.
2. Listen to hospital rounds on at least 4 different days to become familiar with the format of daily rounds and how team members present and discuss patients on rounds.
3. Develop skills in prerounding by identifying relevant electronic health record data to create an oral presentation, as measured by self-reported surveys.
4. Develop skills in delivering oral presentations through structured practice and personalized feedback, aiming to deliver three presentations per week that include an assessment and plan.
5. Develop skills in articulating clinical reasoning in oral presentations, as measured by self-reported surveys.

Citation:
Sukumar S, Zakaria A, Lai CJ, Sakumoto M, Khanna R, Choi N. Designing and implementing a novel virtual rounds curriculum for medical students' internal medicine clerkship during the COVID-19 pandemic. *MedEdPORTAL.* 2021;17:11106.
https://doi.org/10.15766/mep_2374-8265.11106

Introduction

The COVID-19 pandemic has caused widespread, unprecedented challenges in society.[1] Medical education has been particularly affected, as it falls at the intersection of multiple systems that have been individually affected by the pandemic (i.e., health care, education, and economics). Medical schools face the pressure of continuing to train future physicians in order to advance medical student training and produce a workforce that

Copyright © 2021 Sukumar et al. This is an open-access publication distributed under the terms of the Creative Commons Attribution license.

will tackle the pandemic in coming years. Traditionally, medical students spend their first 1-2 years in a classroom environment (preclerkship education), learning from educational strategies such as lectures. The challenges posed by the pandemic to preclerkship education have largely been addressed by implementing online and videoconferencing-based didactics that enable students to remotely meet learning objectives.[2] This transition to a flipped classroom model has even been described as an opportunity to modernize preclerkship education, making learning more student-centered and efficient.[3,4] In contrast, clerkship education is deliberately rooted in the clinical setting, a strategy that taps into the educational theories of cognitive apprenticeship, situated learning, and workplace learning. These theories rely on in-person clinical immersion experiences to facilitate students' learning and interactions with patients and provider teams.

Due to the COVID-19 pandemic, in March 2020 the Association of American Medical Colleges strongly suggested "that medical students not be involved in any direct patient care activities."[5,6] This caused significant disruption to clinical clerkship education, with many schools subsequently transitioning to didactic learning. While there is concern that clerkship education without direct clinical exposure could compromise student experiences and learning, no national guidance currently exists on how to reengage students whose clinical education has been paused due to the pandemic.[1] Some guidance has been sought from medicals schools that previously educated students in the setting of natural disasters or epidemics, including Hurricane Katrina and SARS-CoV-1.[7-9] Many of these schools transitioned to video or audiotaped vignettes and recordings, online chat rooms, and problem-based learning; some even relied upon neighboring unaffected institutions to provide clinical teaching sites.[7,8,10] In the setting of COVID-19, many medical schools have made similar adaptations to clerkship education by shortening the length of clerkships and relying upon online modules and didactics.[11] However, data indicate that while such solutions may not compromise textbook learning, they may produce worse outcomes for clinical education.[12] In addition, prior natural disasters and pandemics were limited in temporal and geographic scope; medical educators today face the unique challenge of educating clerkship students in a global pandemic of unknown duration.[1]

Although recreating and supplementing components of in-person clerkships with simulated patient interactions have been shown to improve medical knowledge and skills, no model effectively replaces the multifaceted experience of working with a medical team and learning from patients in the clinical environment.[13,14] Online case-based learning and simulations may not entirely recreate the experience of making an assessment/plan with evolving data, receiving real-time feedback, and engaging in the care of patients who may not present exactly as depicted in textbooks.[5] While outpatient clinical experiences and individual emergency medicine patient cases have been recreated with telehealth technology, these curricula did not focus on the inpatient rounding experience.[15,16] For instance, a previously described curriculum that provided medical students with a prewritten script and checklist to facilitate individual patient encounters in the emergency room setting did not provide the experiences of continuously caring for a hospitalized patient, developing organically evolving differentials, and managing plans over days as new data became available.[16] In addition, such previously described curricula did not provide students with exposure to the teamwork required when rounding with learners of different levels (i.e., acting interns and residents) or when working with interprofessional team members to optimize care coordination. Although there are models that use technology to individually recreate morning report, bedside teaching, and modified patient encounters, we are not aware of any published literature or *MedEdPORTAL* curricula that describes changes made to a core inpatient internal medicine (IM) clerkship using technology to virtually simulate hospital rounds in the setting of COVID-19.[17-19]

In line with AAMC recommendations, our institution's medical students temporarily did not participate in onsite clinical activities in the spring of 2020. During this time, we devised an innovative way to provide students with clinical exposure to inpatient IM, providing experiential learning opportunities that cannot be replicated solely by online modules and didactics.

Methods

From April to early July 2020, four blocks of students previously scheduled to participate in onsite inpatient IM clerkships were instead required to participate in a 2-week virtual learning period. During this virtual learning period, they learned from a combination of online didactics and case studies, similar to other clerkships' curricular blueprints at our institution. We, the study investigators and IM clerkship leaders, created a novel virtual rounds (VR) curriculum to supplement the IM virtual learning period. We developed VR using the six pillars for medical curricula from Kern's *Curriculum Development for Medical Education*: (1) problem identification, (2) needs assessment of targeted learners, (3) goals and specific measurable objectives, (4) educational strategies, (5) implementation, and (6) evaluation

Copyright © 2021 Sukumar et al. This is an open-access publication distributed under the terms of the Creative Commons Attribution license.

and feedback.[20] Weekly feedback provided by students and tele-instructors was used to iteratively revise VR.

Problem Identification

We deconstructed and identified areas of medical training normally gained on an IM clerkship that would be compromised if students learned only from online didactics, case studies, and simulations (i.e., prerounding, oral presentation skills, diagnostic reasoning, and medical management discussion).

Needs Assessment of Targeted Learners

Targeted learners were four blocks of third-year medical students rotating on a 2-week virtual learning component of their IM clerkship at a tertiary care academic medical center. Some of these students had completed one to two core clerkships in person prior to starting VR, and others had completed only 2-week electives. Areas of improvement based on preliminary feedback from a 1-week pilot of VR included a need for focused teaching on how to effectively navigate the electronic health record (EHR), as well as a need for curriculum flexibility with ample time for resident-led feedback and clinical teaching. Based on this, the VR curriculum guide (Appendix A) was edited to encourage best practices for resident-led rounds during small group, and an orientation to prerounding was added to the curriculum (Appendix B).

Goals and Measurable Objectives

We designed VR to help students develop skills in inpatient prerounding, oral presentation delivery, clinical reasoning, and synthesizing feedback and clinical teaching from multiple physicians. These objectives are detailed in the Educational Objectives.

Educational Strategies

We wanted to simulate cognitive apprenticeship, situated learning, and workplace learning techniques central to hospital rounds. The prerounding component of the curriculum was created by assigning students to preround on hospitalized patients remotely via the EHR. The collaborative work environment of rounds was recreated by asking students to listen to hospital rounds, with subsequent participation in a telemedicine VR small group. Best practices for small-group educational strategies were provided in the VR curriculum (Appendix A).

Implementation of VR

Prior to starting VR, students and tele-instructors were sent a curriculum guide detailing expectations, guidelines, and best practices (Appendix A). Tele-instructors were provided with an additional guide on teaching diagnostic reasoning (Appendix C). Tele-instructors did not provide grades or required evaluations.

Telemedicine VR teams facilitated VR via videoconferencing (Zoom, version 5.0.2) 3 days a week for 2 weeks. Each telemedicine virtual rounding team consisted of two to four (average of three) third-year medical student learners, one IM resident teaching assistant, one fourth-year medical student teaching assistant (TA), and one hospitalist attending physician. We recruited volunteer attendings and TAs by email, requesting they commit to facilitating three 1-hour telemedicine VR small groups during a 1-week period. Several residents and attendings who volunteered were able to do so because they were on flexible rotations or had had their schedules disrupted by the COVID-19 pandemic.

VR had three curricular components: (1) virtual prerounding; (2) formulating a SOAP (subjective, objective, assessment, plan) oral presentation; and (3) giving a SOAP presentation to a VR small group with subsequent feedback and clinical teaching. VR students did not participate in direct patient care.

Virtual prerounding: On the first day of VR, the VR resident TA led an interactive orientation, demonstrating effective navigation of the EHR to obtain pertinent patient information. Resident TAs were provided with a prerounding teaching guide (Appendix B) to assist with this teaching. Each third-year student was assigned to follow one hospitalized patient from the inpatient IM service at a tertiary care hospital. Students were securely emailed the patient's medical record number and contact information for the wards acting intern or intern caring for their patient in the hospital. Each morning of VR, students were asked to spend 30 minutes prerounding on their patient using remote access to the EHR. Students then contacted the wards intern caring for their patient via phone call or videoconferencing and listened to the morning rounds presentation and discussion pertaining to their patient. Students were asked to preround on the same patient each day of VR by reviewing the EHR and calling into rounds. If their patient was discharged, students were assigned a new hospitalized patient to follow. Most students followed one unique patient each week.

Formulating a SOAP presentation: Students then created a 5- to 10-minute SOAP presentation on their patient. They were encouraged to include physical exam findings learned from hospital rounds and to think of additional examination maneuvers or questions they would have asked the patient to augment their clinical reasoning and diagnostic workup. They were

Copyright © 2021 Sukumar et al. This is an open-access publication distributed under the terms of the Creative Commons Attribution license.

also encouraged to use resources such as UpToDate software (Wolters Kluwer) and PubMed (https://pubmed.ncbi.nlm.nih.gov/) to learn about unfamiliar diseases or medical management and to include citations in their presentations.

Telemedicine VR small group: Students delivered their SOAP presentations to their telemedicine VR team during small group via videoconferencing. They received feedback on their presentation from the fourth-year medical student TA, followed by feedback from the resident TA. The attending was encouraged to keep time and contribute additional feedback when needed. TAs were encouraged to teach relevant clinical topics and diagnostic reasoning, similar to chalk talks or teaching done on hospital rounds. TAs and attendings were encouraged to summarize daily learning points and reference materials via email or shared online document.

Evaluation and Feedback for the VR Curriculum

We developed a student survey (Appendix D) and a tele-instructor survey (Appendix E) with Likert-scale and open-ended questions to (1) evaluate the practicality and efficacy of VR, (2) identify areas for curriculum improvement, and (3) understand what, if any, role such a novel curriculum might have in the IM clerkship after COVID-19. We designed the survey questions to reflect curricular educational objectives. We piloted the surveys for clarity and usability with medical students and faculty not participating in the curriculum. After students completed the VR curriculum, we emailed an invitation to students, TAs, and attendings to fill out the survey within Qualtrics (version 2020). Survey data were collected anonymously, and students were informed that survey participation would not be graded. This study was deemed exempt by the Institutional Review Board of the University of California, San Francisco.

Results

From April 23 to July 3, 2020, four blocks of students completed the VR curriculum. Data on VR and survey participation are shown in Table 1. Twenty-nine clerkship students and 34 volunteer tele-instructors (11 attendings, 12 resident TAs, and 11 fourth-year medical student TAs) participated in VR. Student survey response rate was 14 out of 29 (48%). Tele-instructor response rate was 25 out of 34 (74%).

Table 1. Participant Breakdown and Response to Survey

Type of VR Participant	No. of VR Participants	No. (%) of Survey Participants
Third-year medical student	29	14 (48)
Fourth-year medical student TA	11	7 (64)
Resident TA	12	10 (83)
Attending	11	8 (73)

Abbreviations: TA, teaching assistant; VR, virtual rounds.

Student Responses

Overall perception of curriculum value: Data on student perception of changes in clinical abilities and confidence due to VR are shown in Table 2. The majority of student respondents perceived improvement in their ability to preround (86%), give an oral presentation (93%), and clinically reason (61%). Ninety-three percent felt more confident in their ability to succeed in their future in-person IM clerkship. In open-ended comments about the most valuable aspects of the curriculum, several students noted that it was "very helpful learning how to go through EHR and… interpret and incorporate patient history, data, labs and imaging into A/P [assessment and plan]." Students highlighted the importance of small group as "a space to rehearse [oral presentations] and work out small matters of style that you might not realize go into rounding otherwise." Some noted that learning from a fourth-year medical student was especially engaging and valuable. Others appreciated clinical teaching. Several students noted that curriculum "flexibility was… really important so that each group could develop their own structure."

Value of learning with real patients: In open-ended comments about the value of learning with real patients in the VR curriculum compared to learning from prepared (didactic) cases, multiple students found following a real patient to be "more interactive," "more engaging," and a greater "investment into learning." Although one student remarked that VR was "significantly less efficient" than learning from case studies, another student noted, "Following a real patient was one million times more interesting and engaging than prepared cases! It felt like I was

Table 2. Student Perception of Changes to Clinical Abilities and Confidence

Survey Item	No. (%)				
	Much Worse	Somewhat Worse	About the Same	Somewhat Better	Much Better
Ability to preround	0 (0)	0 (0)	2 (14)	7 (50)	5 (36)
Ability to give an oral presentation	0 (0)	0 (0)	1 (7)	8 (57)	5 (36)
Ability to clinically reason	0 (0)	0 (0)	5 (38)	5 (38)	3 (23)
Confidence approaching the clinical component of internal medicine clerkship	0 (0)	0 (0)	1 (7)	9 (64)	4 (29)

Copyright © 2021 Sukumar et al. This is an open-access publication distributed under the terms of the Creative Commons Attribution license.

on the wards and responsible for my patient except the focus was only my learning.” Students reported that while they were not involved in patient care, it was rewarding “to see patients get better in real time and problem solve in real time” and that they were still exposed to “more practical things… like discharge goals and how to balance a lot of problems at once.” Several students “enjoyed the granularity and attention to detail required when thinking about a real patient’s hospital course as opposed to online cases.” While some noted that real patients were “much more complicated, [and] difficult to generalize from, [they were] good in terms of working through a wide differential and sorting data.” Students found VR to be “more memorable learning” and “SO much better” than learning from prepared cases; one student wished they could have VR for every clerkship.

Evaluation of curricular elements: Data on student perception of different curricular elements are shown in Table 3. Ninety-three percent of students found that the curricular aspects focusing on how to preround were useful (moderately to extremely useful). Specifically, they highly rated the following elements: practicing how to use the EHR to identify relevant data for rounding, learning how to incorporate new data into oral presentations, and learning how to incorporate medical resources into oral presentations. Seventy-nine percent reported that listening to hospital rounds was useful. All students (100%) found the telemedicine VR small groups to be useful. Students reported that delivering SOAP presentations, receiving feedback on their presentations, and practicing how to incorporate feedback into subsequent presentations were particularly useful.

Incorporation of technology into the curriculum: Seventy-nine percent found videoconferencing to be a good or excellent tool to achieve curriculum educational objectives. Forty-three percent reported their small group used screen-sharing technology to supplement learning by facilitating journal article dissemination, chalk talks, or PowerPoints. The majority of students (57%) reported using a shared document or email thread to maintain a record of learning points.

Areas identified for curriculum improvement: Nearly half of the respondents reported poor or very poor ability to hear hospital rounds presentations, with two students suggesting removing the requirement for students to call into hospital rounds. Two students commented that time constraints limited their ability to maximize small-group learning.

Tele-instructor Responses

Perception of ability to teach and identification of unique learning opportunities through VR: Data on tele-instructor perception of the curriculum are shown in Table 4. Compared to in-person rounds, 92% of tele-instructor respondents felt that VR offered as good, if not better, opportunities to provide medical student level clinical teaching. Sixty percent felt that VR enabled them to provide more feedback on oral presentation skills. Over two-thirds of tele-instructors felt that their overall ability to teach on VR was better than during in-person rounds. Nearly a quarter

Table 3. Student Perception of Usefulness of Virtual Rounds Curricular Components

	No. (%)				
**Survey Item**	**Not at All Useful**	**Slightly Useful**	**Moderately Useful**	**Very Useful**	**Extremely Useful**
Prerounding	0 (0)	1 (7)	3 (22)	3 (21)	7 (50)
Practicing how to use the electronic health record to identify relevant patient data for rounds	0 (0)	2 (14)	1 (7)	5 (36)	6 (43)
Developing an approach to chart reviewing	1 (7)	1 (7)	3 (22)	2 (14)	7 (50)
Incorporating new labs/imaging/results into your presentation	2 (14)	2 (14)	0 (0)	5 (36)	5 (36)
Incorporating resources such as literature searches or UpToDate.com into your presentation	3 (22)	1 (7)	2 (14)	7 (50)	1 (7)
Listening to hospital rounds	1 (7)	2 (14)	5 (36)	3 (22)	3 (21)
Listening to a subintern or intern’s presentation	0 (0)	3 (21)	3 (21)	4 (29)	4 (29)
Listening to feedback on the subintern or intern’s presentation	1 (7)	2 (14)	5 (36)	4 (29)	2 (14)
Exposure to patient-related discussions that occur on rounds	1 (7)	2 (14)	5 (36)	5 (36)	1 (7)
Virtual rounds small group	0 (0)	0 (0)	2 (14)	6 (43)	6 (43)
Following a patient’s clinical course	0 (0)	1 (7)	3 (22)	7 (50)	3 (21)
Giving SOAP or assessment and plan presentations	0 (0)	0 (0)	3 (21)	2 (14)	9 (65)
Receiving feedback on oral presentations	0 (0)	1 (8)	1 (8)	3 (23)	8 (61)
Practicing how to incorporate feedback into subsequent oral presentations	0 (0)	0 (0)	1 (8)	4 (31)	8 (61)
Practicing skills in clinical reasoning (including problem representation, developing differentials)	0 (0)	0 (0)	5 (36)	4 (28)	5 (36)
Listening to oral presentations given by peers	0 (0)	0 (0)	5 (56)	3 (33)	1 (11)
Listening to feedback given to peers on their oral presentations	0 (0)	0 (0)	2 (22)	6 (67)	1 (11)
Advancing clinical knowledge	0 (0)	0 (0)	5 (36)	3 (21)	6 (43)

Abbreviation: SOAP, subjective, objective, assessment, plan.

Copyright © 2021 Sukumar et al. This is an open-access publication distributed under the terms of the Creative Commons Attribution license.

Table 4. Tele-instructor Perceptions of Ability to Teach Through Virtual Rounds, Compared to Hospital Rounds

	No (%)				
**Survey Item**	**Much Worse**	**Somewhat Worse**	**About the Same**	**Somewhat Better**	**Much Better**
Opportunities to provide feedback on oral presentation skills	0 (0)	3 (12)	7 (28)	4 (16)	11 (44)
Opportunities to provide feedback on medical management discussed in assessment and plan	1 (4)	8 (32)	8 (32)	5 (20)	3 (12)
Opportunities to provide medical student–level clinical teaching	0 (0)	2 (8)	9 (36)	5 (20)	9 (36)
Overall ability to teach	0 (0)	0 (0)	8 (35)	10 (43)	5 (22)

of tele-instructors appreciated that VR facilitated dedicated teaching time for clerkship students without competing demands of hospital rounds, such as resident education or patient care. One tele-instructor appreciated not being required to evaluate student and felt more comfortable providing "nitpicky" feedback to help students improve on small details.

Perception of student learning: Ninety-two percent felt that VR catalyzed students' improvement in both the organization and the efficiency of their oral presentations. Consistent with student self-reports, 92% of tele-instructors felt that students were more confident in giving oral presentations by the end of the VR curriculum. Sixty percent of tele-instructors believed that by the end of VR, students conveyed improved diagnostic reasoning and clinical knowledge in oral presentations. One tele-instructor summarized, "It's hard to overstate the benefit of actually seeing and examining a patient every day. That said, I think this was a highly valuable substitute… given the limitations… afforded by the pandemic."

Personal impact of teaching: All tele-instructors felt they personally benefited from teaching VR (through organizing formal chalk talks, learning how to provide constructive feedback, and increasing their own medical knowledge). Eighty-eight percent felt that teaching VR enhanced their interests in medical education. Nearly all (96%) tele-instructors said they would likely volunteer to teach VR again if their schedule permitted. Multiple tele-instructors commented that VR brought them "joy" and that they "missed" and "loved" teaching clerkship students. Fourth-year medical student TAs noted that teaching allowed them to "reflect on [their] growth" as a student and prepared them for fourth-year clinical rotations. Residents and attendings enjoyed seeing "engaged" third-year students quickly improve and gain confidence. They also enjoyed seeing fourth-year medical student TAs take on a teaching role.

Role of VR in clerkships after COVID-19: All tele-instructors felt there could be a role for VR to remain part of the IM clerkship even after COVID-19. One tele-instructor noted,

> Moving forward, I see a role for VR to be used in preclinical [second-year medical students] as a means to transition from the preclinical year to the medical wards so that they can come in with a solid foundation for oral presentation skills and a systematic approach to prerounding.

Another remarked,

> It'd be nice if there were more time made inpatient for teaching [third-year medical students] how to present instead of starting in on the medical reasoning from day one. VR might be a way for that to happen if people are too busy in the hospital.

One tele-instructor commented that VR provided "time to [regularly] focus on presentations outside normal hospital rounds.… This could be done regularly by… attendings in a non-COVID era."

Areas identified for curriculum improvement: While some tele-instructors appreciated being able to teach and provide feedback to multiple students at once so that students could learn from the feedback given to others, other tele-instructors would have preferred to provide feedback in private one-on-one sessions. Sixteen percent of tele-instructors felt rushed having three medical students in a 1-hour time slot for VR small group and would have preferred having two students.

There was no significant difference in these responses based on the type of tele-instructor surveyed (fourth-year medical student TA, resident TA, or attending).

Discussion

We have reported on an innovative and iteratively revised VR curriculum that sought to fill a gap in clerkship education resulting from the COVID-19 pandemic. In particular, we used technology to simulate the experience of prerounding on a clinically evolving patient and subsequently presenting in a team-based environment similar to hospital rounds.

Copyright © 2021 Sukumar et al. This is an open-access publication distributed under the terms of the Creative Commons Attribution license.

The COVID-19 pandemic's pressure on the medical system has exposed and exacerbated preexisting gaps in medical education and forced a reevaluation of many core principles of clinical education. Students and tele-instructors both highlighted that the VR curriculum filled a gap in providing increased time for direct and detailed feedback on presentation skills without competing demands of patient care, compared to traditional clerkships. This need for increased one-on-one teaching in the traditional clinical environment has been previously suggested by other curricula implemented in the setting of COVID-19, and our resource offers a way in which virtual learning can help fill this gap.[16] We have also learned the importance of formally teaching students how to preround, a skill usually learned through workplace learning strategies during in-person clerkships. Providing a formal orientation guide to prerounding and protected time for learning how to navigate the EHR was greatly valued by students and another effective use of virtual learning.

Telemedicine VR small groups recreated elements of situated learning found in hospital rounds by facilitating academic discussion between learners and instructors of different levels. Elements of cognitive apprenticeship and workplace learning were replicated by having students listen to hospital rounds; some students even noted that this helped them learn about patient care tasks such as discharge planning. However, there were limitations to the format of VR. VR did not include physical exam or direct patient care, the latter of which is core to the theories of cognitive apprenticeship and workplace learning. In addition, while calling into hospital rounds provided opportunities to listen to different ways of framing clinical problems, this component of the curriculum was hindered by logistic and technical difficulties and was less useful to students. Tele-instructors found that teaching about medical management on VR was difficult, compared to providing feedback on oral presentations and diagnostic workup, and some would have preferred to focus the limited time available in small group on oral presentation and diagnostic skills. A limitation of the study was the lack of a direct comparison group since all students assigned to the site were required to participate in the VR pilot program (there were no in-person clerkship students who could serve as controls). Another limitation was that only 48% of students who participated in VR responded to our research survey. This low response rate may have been due to general pandemic-related stress, competing academic demands for student time (i.e., studying for clerkship shelf exams), or survey fatigue given that our research survey was optional and students were simultaneously required to respond to mandatory end-of-course survey evaluations.

Despite these limitations, student and tele-instructor respondents found VR to be personally rewarding and an effective model to facilitate clerkship clinical teaching while maintaining social distancing guidelines. In an uncertain time when third-year medical student education could have been perceived as a burden on an already struggling health care system, it was reassuring to see strong support from volunteer near-peers (fourth-year students), residents, and attendings who taught VR and reaffirmed their dedication to, and passion for, teaching in their survey comments. Several tele-instructors noted that they wished they themselves could have participated in a similar curriculum before beginning on the wards, and some students wished such a curriculum was available for other core clerkships.

Given the unknowns with the current pandemic, it is possible that future clerkship students will have to complete parts of their clerkship education remotely with distance learning. While the VR curriculum at our institution was fortunate to have the support of many volunteer physicians, the VR model could be replicated on a larger scale with more students and fewer tele-instructors by having only one physician educator per small group. The time commitment from participating physicians in such a model would still be low (1 hour per small group), and this would be a practical modification to VR. In addition, asynchronous learning could be built into such curricula. By using GoogleDocs and shared online resources, tele-instructors could summarize teaching points and answer student questions, so that additional learning could happen outside of the dedicated 1-hour small group. Another variation on this curriculum could focus on specific educational elements (i.e., teaching only prerounding or presentation skills). Since 93% of students thought VR made them more confident as they approached the clinical component of their medicine clerkship, a future role for the VR curriculum in the non-COVID setting might be as a virtual immersion in the clinical environment or a particular clerkship, during which preclerkship students would learn prerounding and oral presentation skills. Such a curriculum could even be facilitated by near-peers, such as fourth-year medical students, and would not require resident or attending participation.

The pressure to change our approach to medical education in the setting of COVID-19 could prove to be an opportunity for creative reforms and improvements. A greater reliance on technology, for instance, might turn out to be a beneficial improvement to medical education. Telemedicine has allowed some outpatient rotations to continue in the setting of COVID-19 without compromising the quality of education.[15] Some medical schools have even been able to use real-time videoconferencing

Copyright © 2021 Sukumar et al. This is an open-access publication distributed under the terms of the Creative Commons Attribution license.

to simulate morning report, bedside teaching, and modified patient encounters.[17-19] One program developed a curriculum that, through supplemental virtual chart review requirements and virtual case presentations to faculty, resulted in students and faculty having increased opportunities for practice of clinical reasoning skills and one-on-one teaching time.[16] It is unlikely that any remote form of education will replace the multifaceted education provided by in-person clerkships. However, this novel VR curriculum demonstrates the utility of formal virtual prerounding and rounding education to supplement an IM clerkship curriculum at a crucial time when in-person learning is not possible. Based on positive feedback from students and tele-instructors, the VR curriculum model can be an effective tool for providing educational virtual clinical experiences during the COVID-19 pandemic. This curriculum, as well as the lessons learned from its design and implementation, may continue to have a role in supplementing medical education even after in-person clerkships resume.

Appendices

A. VR Curriculum Guide.docx

B. Virtual Rounds Orientation Guide.docx

C. Diagnostic Reasoning Terms and Pitfalls.docx

D. Student Survey.docx

E. Tele-instructor Survey.docx

All appendices are peer reviewed as integral parts of the Original Publication.

Smrithi Sukumar: Medical Student, University of California, San Francisco, School of Medicine; ORCID: https://orcid.org/0000-0003-0356-4875

Adam Zakaria: Medical Student, University of California, San Francisco, School of Medicine

Cindy J. Lai, MD: Professor, Department of Medicine, and Director, Internal Medicine Clerkship, University of California, San Francisco, School of Medicine

Matthew Sakumoto, MD: Assistant Professor–Volunteer, Department of Medicine, University of California, San Francisco, School of Medicine

Raman Khanna, MD, MAS: Associate Professor, Department of Medicine, University of California, San Francisco, School of Medicine

Nancy Choi, MD: Assistant Professor, Department of Medicine, and Assistant Site Director, Internal Medicine Clerkship, University of California, San Francisco, School of Medicine

Acknowledgments
We thank our University of California, San Francisco, clerkship students and volunteer fourth-year students, residents, and attendings for participating in the curriculum and providing their feedback. We also appreciate the feedback from educational leaders in the School of Medicine and Department of Medicine and from the Division of Hospital Medicine's Incubator Research Group.

Disclosures
Raman Khanna reports that CareWeb, a product he developed, has been licensed by Hillrom. That product is unrelated to the current work.

Funding/Support
None to report.

Ethical Approval
The University of California, San Francisco, Institutional Review Board approved this study.

References

1. Akers A, Blough C, Iyer MS. COVID-19 implications on clinical clerkships and the residency application process for medical students. *Cureus*. 2020;12(4):e7800. https://doi.org/10.7759/cureus.7800

2. Almarzooq ZI, Lopes M, Kochar A. Virtual learning during the COVID-19 pandemic: a disruptive technology in graduate medical education. *J Am Coll Cardiol*. 2020;75(20):2635-2638. https://doi.org/10.1016/j.jacc.2020.04.015

3. Chen CH, Mullen AJ. COVID-19 can catalyze the modernization of medical education. *JMIR Med Educ*. 2020;6(1):e19725. https://doi.org/10.2196/19725

4. Newman NA, Lattouf OM. Coalition for medical education—a call to action: a proposition to adapt clinical medical education to meet the needs of students and other healthcare learners during COVID-19. *J Card Surg*. 2020;35(6):1174-1175. https://doi.org/10.1111/jocs.14590

5. Ferrel MN, Ryan JJ. The impact of COVID-19 on medical education. *Cureus*. 2020;12(3):e7492. https://doi.org/10.7759/cureus.7492

6. Association of American Medical Colleges. *Interim Guidance on Medical Students' Voluntary Participation in Direct Patient Contact Activities: Principles and Guidelines*. Liaison Committee on Medical Education. March 23, 2020. Accessed December 27, 2020. https://lcme.org/wp-content/uploads/filebase/March-23-2020-Interim-Guidance-on-Medical-StudentsE28099-Voluntary-Participation-in-Direct-Patient-Contact-Activities.pdf

7. Krane NK, Kahn MJ, Markert RJ, Whelton PK, Traber PG, Taylor IL. Surviving Hurricane Katrina: reconstructing the educational enterprise of Tulane University School of Medicine. *Acad Med*. 2007;82(8):757-762. https://doi.org/10.1097/ACM.0b013e3180cf6ee5

Copyright © 2021 Sukumar et al. This is an open-access publication distributed under the terms of the Creative Commons Attribution license.

8. Patil NG, Chan YHY. SARS and its effect on medical education in Hong Kong. *Med Educ*. 2003;37(12):1127-1128. https://doi.org/10.1046/j.1365-2923.2003.01723.x
9. Clark J. Fear of SARS thwarts medical education in Toronto. *BMJ*. 2003;326(7393):784. https://doi.org/10.1136/bmj.326.7393.784/c
10. Lim ECH, Oh VMS, Koh DR, Seet RCS. The challenges of "continuing medical education" in a pandemic era. *Ann Acad Med Singap*. 2009;38(8):724-726.
11. Calhoun KE, Yale LA, Whipple ME, Allen SM, Wood DE, Tatum RP. The impact of COVID-19 on medical student surgical education: implementing extreme pandemic response measures in a widely distributed surgical clerkship experience. *Am J Surg*. 2020; 220(1):44-47. https://doi.org/10.1016/j.amjsurg.2020.04.024
12. Crawford BE, Kahn MJ, Gibson JW, Daniel AJ, Krane NK. Impact of Hurricane Katrina on medical student academic performance: the Tulane experience. *Am J Med Sci*. 2008;336(2):142-146. https://doi.org/10.1097/MAJ.0b013e318180f1b7
13. Ali J, Al Ahmadi K, Williams JI, Cherry RA. The standardized live patient and mechanical patient models—their roles in trauma teaching. *J Trauma*. 2009;66(1):98-102. https://doi.org/10.1097/TA.0b013e318193ba84
14. Gillett B, Peckler B, Sinert R, et al. Simulation in a disaster drill: comparison of high-fidelity simulators versus trained actors. *Acad Emerg Med*. 2008;15(11):1144-1151. https://doi.org/10.1111/j.1553-2712.2008.00198.x
15. Goldenberg MN, Hersh DC, Wilkins KM, Schwartz ML. Suspending medical student clerkships due to COVID-19. *Med Sci Educ*. 2020;30(3):1273-1276. https://doi.org/10.1007/s40670-020-00994-1
16. Chandra S, Laoteppitaks C, Mingioni N, Papanagnou D. Zooming-out COVID-19: virtual clinical experiences in an emergency medicine clerkship. *Med Educ*. 2020;54(12): 1182-1183. https://doi.org/10.1111/medu.14266
17. Murdock HM, Penner JC, Le S, Nematollahi S. Virtual Morning Report during COVID-19: a novel model for case-based teaching conferences. *Med Educ*. 2020;54(9):851-852. https://doi.org/10.1111/medu.14226
18. Hofmann H, Harding C, Youm J, Wiechmann W. Virtual bedside teaching rounds with patients with COVID-19. *Med Educ*. 2020;54(10):959-960. https://doi.org/10.1111/medu.14223
19. Tsang ACO, Lee PP, Chen JY, Leung GKK. From bedside to webside: a neurological clinical teaching experience. *Med Educ*. 2020;54(7):660. https://doi.org/10.1111/medu.14175
20. Thomas PA, Kern DE, Hughes MT, Chen BY, eds. *Curriculum Development for Medical Education: A Six-Step Approach*. 3rd ed. Johns Hopkins University Press; 2016.

Received: July 29, 2020
Accepted: November 24, 2020
Published: March 2, 2021

Copyright © 2021 Sukumar et al. This is an open-access publication distributed under the terms of the Creative Commons Attribution license.
